# Mid-Term Outcomes and Echocardiographic Predictors of TTVR: Insights from the TRAVEL Study

**DOI:** 10.3390/jcdd13080381

**Published:** 2026-08-11

**Authors:** Xing Zhao, Hao Shi, Ying Peng, Xin Meng, Jian Yang, Yutong Jiang, Fanglin Lu, Xiaopeng Hu, Kunjing Pang

**Affiliations:** 1 Department of Echocardiography, Fuwai Hospital, National Center for Cardiovascular Disease, Chinese Academy of Medical Science and Peking Union Medical College, Beijing 100037, China; 2 Department of Cardiovascular Surgery, Fuwai Hospital, National Center for Cardiovascular Disease, Chinese Academy of Medical Science and Peking Union Medical College, Beijing 100037, China; 3 Department of Cardiology, West China Hospital, Sichuan University, Chengdu 610041, China; 4 Department of Ultrasound, Xijing Hospital, Air Force Medical University, Xi’an 710032, China; 5 Department of Cardiovascular Surgery, Xijing Hospital, Air Force Medical University, Xi’an 710032, China; 6 Department of Cardiovascular Surgery, Shanghai General Hospital, Shanghai Jiaotong University, Shanghai 200080, China

**Keywords:** transcatheter tricuspid valve replacement, prognosis, predictors, echocardiography

## Abstract

Background: The mid-term prognosis after transcatheter tricuspid valve replacement (TTVR) is poorly defined, and echocardiographic predictors remain uncertain. Objectives: To describe mid-term all-cause mortality, 30-day major adverse events (MAEs), and echocardiographic risk markers of TTVR. Methods: Consecutive patients undergoing TRAVEL (Transcatheter right atrial-ventricular valve replacement With LuX-Valve) at three centers were retrospectively analyzed. Firth Cox regression was applied for mortality, and Firth logistic regression was used for exploratory MAE analyses. Results: A total of 62 patients (median age 66.5 years [61.0–72.8], 83.8% female) were included. Over a mean follow-up of 46.2 months, seven deaths (11.2%) occurred, corresponding to mid-term survival of 88.8%. Within 30 days, 14 patients (22.6%) experienced MAEs, including three deaths (4.8%), major bleeding in eight (12.9%), surgical re-exploration in seven (11.3%), repeat open-heart tricuspid valve replacement in one (1.6%), and permanent pacemaker implantation in six (9.7%). TR was reduced to mild or less in 95.2% at 1 year. Conclusions: In this LuX-Valve TTVR cohort, 30-day MAEs mainly reflected perioperative or device-related complications rather than a direct signal of baseline RV dysfunction or remodeling. LuX-Valve implantation showed favorable mid-term survival and clinically meaningful procedural effectiveness; RV-centered echocardiographic markers should be considered hypothesis-generating and require validation in larger cohorts.

## 1. Introduction

Severe tricuspid regurgitation (TR) is associated with reduced survival and impaired quality of life [1]. While conventional surgical repair or replacement is an option, isolated tricuspid valve surgery carries a high perioperative risk, with in-hospital mortality rates ranging from 8% to 10%, particularly in patients with prior left-sided valve surgery, advanced heart failure, or end-organ dysfunction [2,3]. Conventional surgical repair or replacement carries high perioperative risk, and transcatheter tricuspid valve replacement (TTVR) has therefore emerged as a promising alternative for high-risk patients [4,5,6,7]. The therapeutic landscape now includes edge-to-edge repair systems, such as the TriClip device, which has demonstrated safety and efficacy in reducing TR [8], and various orthotopic replacement systems like TRICVALVE, designed to mimic surgical valve replacement via a percutaneous approach [9]. Although early feasibility studies have demonstrated procedural success and symptomatic improvement, data on mid- to long-term outcomes after TTVR remain limited. Transthoracic echocardiography (TTE) represents the cornerstone imaging modality for evaluating eligibility for TTVR and remains the primary tool for follow-up [10,11,12]. However, the prognostic value of echocardiographic parameters in patients undergoing TTVR has not been well validated.

In this study, we analyzed echocardiographic data and follow-up outcomes from three of the eight centers participating in the TRAVEL (Transcatheter Right Atrial-Ventricular Valve Replacement With LuX-Valve) trial, which investigated the LuX-Valve system in patients with severe TR [7]. The objective was to describe mid-term mortality, 30-day MAE characteristics, perioperative complications, procedural effectiveness, and TR reduction associated with LuX-Valve implantation, while exploring RV-focused echocardiographic markers in a hypothesis-generating manner.

## 2. Materials and Methods

### 2.1. Study Population

**Figure 1:**
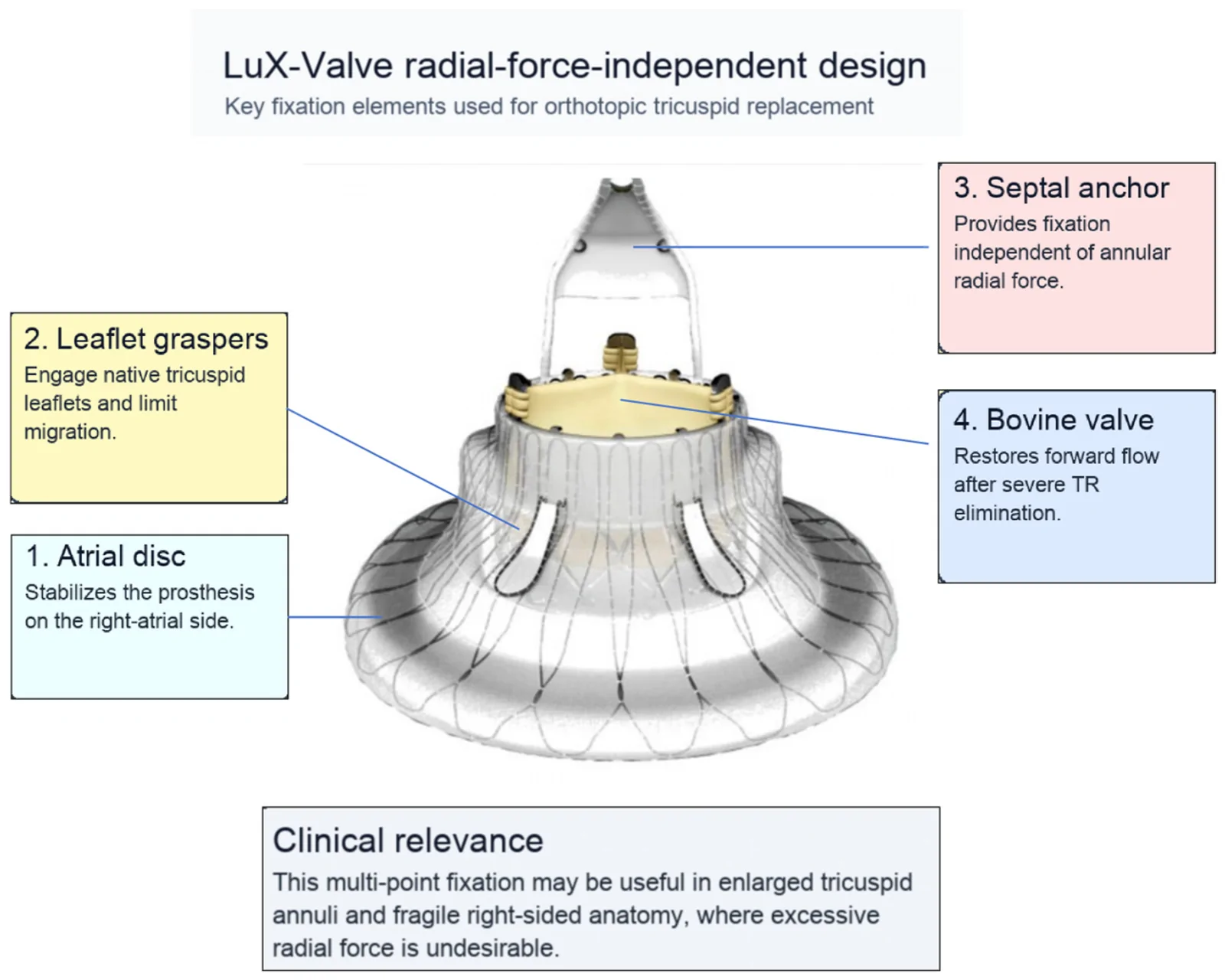
LuX-Valve device design. The radial force-independent system uses an atrial disc, tricuspid leaflet graspers, a bovine pericardial valve, and an interventricular septal anchor to achieve orthotopic fixation without relying primarily on annular radial force.

**Figure 2:**
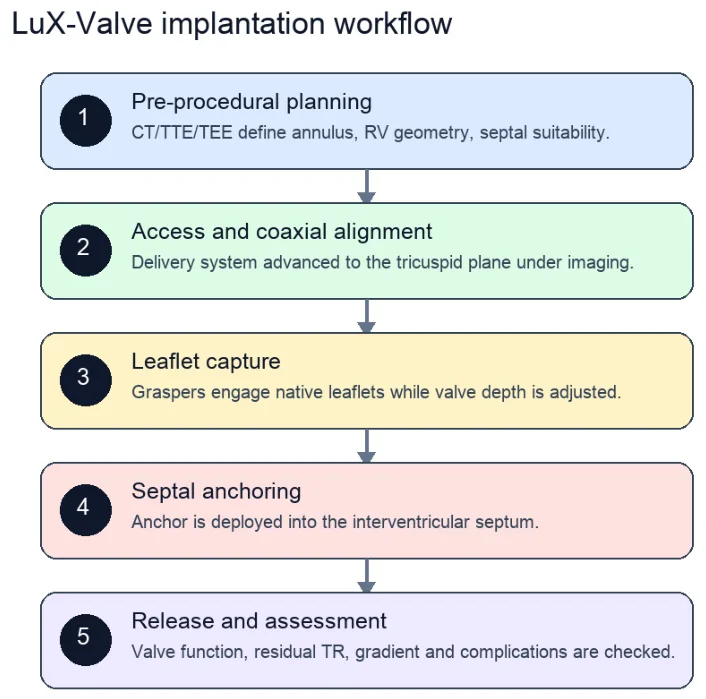
Key steps for LuX-Valve implantation. Pre-procedural imaging defines eligibility and geometry; implantation proceeds through access and coaxial alignment, leaflet capture, septal anchoring, release, and immediate assessment of residual TR, gradients, and complications.

We retrospectively evaluated consecutive patients with severe tricuspid regurgitation who underwent TTVR with the LuX-Valve system and achieved complete follow-up at three participating centers in the TRAVEL trial [7]. No patients were excluded due to missing data. Patients were included if they had symptomatic TR (grade ≥ 4) [13,14] despite optimal medical therapy and were considered at high or prohibitive risk for conventional surgery by a multidisciplinary heart team. The LuX-Valve system is a novel radial force-independent orthotopic transcatheter tricuspid valve (Figure 1 and Figure 2). It consists of a self-expanding nitinol alloy stent frame with two expandable polytetrafluoroethylene-coated discs and a tri-leaflet bovine pericardial tissue valve. A detailed description and illustration of the device and procedural workflow have been previously published [7]. This study has been approved by the Ethics Review Committee of Fuwai Hospital.

### 2.2. Echocardiography

**Figure 3:**
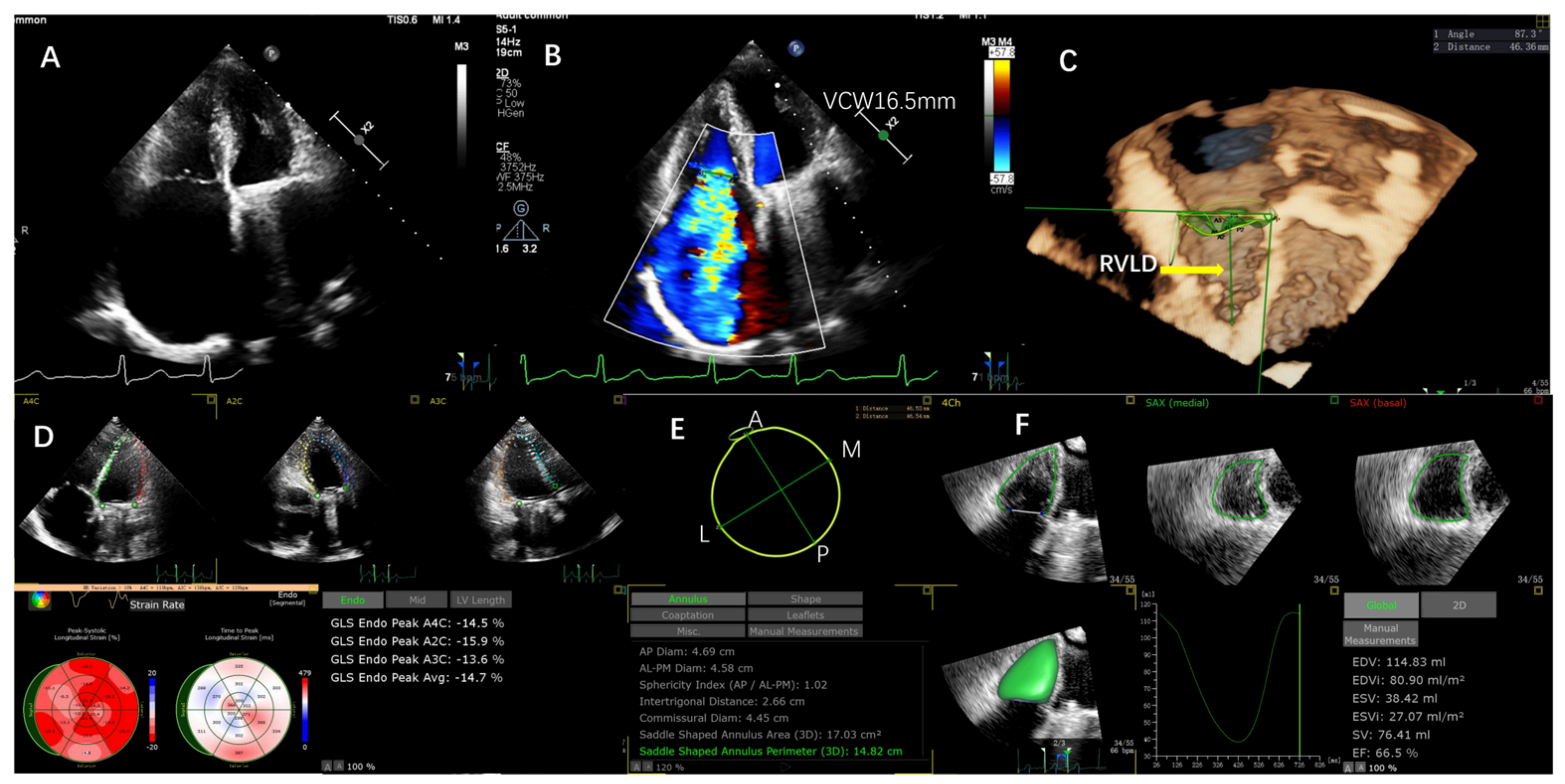
Two-dimensional and three-dimensional echocardiographic measurements. (**A**). Apical four-chamber view of a patient 21 years after mitral valve replacement; (**B**). Tricuspid regurgitation graded as 5, with a vena contracta width (VCW) of 16.5 mm; (**C**). Right ventricular longitudinal diameter (RVLD) measured using three-dimensional echocardiography; (**D**). Strain analysis used for ventricular functional assessment; (**E**). Tricuspid valve anteroposterior diameter (TVAPD) measured using a three-dimensional software package; (**F**). Right ventricular end-systolic volume index (RVESVI) measured using a 3D automated RV analysis software package.

Baseline two-dimensional and three-dimensional transthoracic echocardiography (TTE) was performed within one week prior to TTVR in accordance with current guideline recommendations [13]. Examinations were conducted using Epiq 7c or CvX systems (Philips, Amsterdam, The Netherlands) or the E95 system (GE Vingmed Ultrasound AS, Horten, Norway). All echocardiographic analyses were conducted offline in the core laboratory by an experienced echocardiographer blinded to clinical outcomes, utilizing a CPA 2.5 workstation (TomTec ARENA, Unterschleissheim, Germany). Values were averaged over five consecutive cardiac cycles. Interobserver and intraobserver reproducibility was evaluated in a random subset of 20 patients by calculating intraclass correlation coefficients (ICC). RVFWS was derived from three segments of the RV free wall. RV end-systolic volume was quantified using three-dimensional TTE and indexed to body surface area (RVESVI) (Figure 3). Additional RV-focused parameters included TR grade [14], right atrial volume, RVLD, TVAPD, tricuspid annular plane systolic excursion (TAPSE), RV fractional area change (RV-FAC), tissue Doppler systolic velocity (S-TDI), systolic pulmonary artery pressure (sPAP), and RV-pulmonary artery coupling assessed by TAPSE/sPAP [15,16]. Left-sided parameters were retained only for baseline characterization.

### 2.3. Endpoints

The primary endpoint was 30-day MAE, defined as a composite of cardiovascular death and device-related complications, including significant intraoperative or postoperative hemorrhage, surgical re-exploration via sternotomy, unplanned reintervention, and implantation of a permanent pacemaker for newly developed atrioventricular conduction block. The secondary endpoint was all-cause mortality assessed through 5 years after the index procedure. Given the heterogeneity of the composite MAE endpoint and the limited number of events, individual components were summarized descriptively rather than modeled separately.

### 2.4. Statistical Analysis

Continuous variables are presented as mean ±standard deviation or median [interquartile range], and categorical variables as counts and percentages. Variables for multivariable models were pre-specified based on clinical importance and significance in univariable analysis (*p* < 0.10), with emphasis on RV parameters including RVESVI, RVLD, TVAPD, RVFWS, TAPSE, RV-FAC, S-TDI, sPAP, and TAPSE/sPAP. To minimize overfitting given the small event count, only a maximum of two covariates were included in any multivariable model. Given the limited number of events, Firth’s penalized likelihood method was applied to reduce small-sample bias. Long-term all-cause mortality was analyzed using Firth Cox proportional hazards regression, with results expressed as hazard ratios (HR) and 95% confidence intervals (CI). The proportional hazards assumption was verified using Schoenfeld residuals. In addition, 30-day MAEs were evaluated using Firth logistic regression, with odds ratios (OR) and 95% CI reported. Model performance was assessed by discrimination and calibration. Discrimination was evaluated using time-dependent receiver operating characteristic curves and Harrell’s concordance index. For MAE models, area under the ROC curve was compared using the DeLong test, and calibration was examined graphically with calibration plots. Risk stratification thresholds were prespecified based on clinical relevance and ROC analysis: RVESVI > 42 mL/m^2^ and sPAP > 35, >45, and >50 mmHg. Kaplan–Meier curves were constructed for these subgroups, and differences were compared using the log-rank test. All statistical analyses were performed using R software (version 4.3.2; R Foundation for Statistical Computing, Vienna, Austria). A two-sided *p* value < 0.05 was considered statistically significant. Reviewer-requested exploratory threshold analyses were also performed for TAPSE, RVFWS, 3D RVEF, RV-FAC, and RV basal diameter; tissue Doppler S’ thresholds were not modeled because S’ values were not available in the analyzable dataset.

## 3. Results

### 3.1. Baseline Characteristics

A total of 62 patients with TR grade ≥ 4 were included. The median age was 66.5 years (IQR, 61.0–72.8), and 52 patients (83.8%) were female. All patients were symptomatic, classified as New York Heart Association (NYHA) functional class III (80.6%) or IV (19.4%). Atrial fibrillation was present in 58 patients (93.6%), hypertension in 16 (25.8%), and ascites in five (8.1%). Twenty-six patients (41.2%) had a history of mitral valve replacement, 13 (21.0%) had undergone combined aortic and mitral valve replacement, and six (9.6%) had received permanent pacemaker implantation. The remaining patients were diagnosed with heart failure with preserved ejection fraction (HFpEF, *n* = 12). A detailed breakdown of prior cardiac surgery types is provided in the footnote of Table 1.

Baseline TTE demonstrated preserved RV-FAC and RVFWS, whereas both left atrial and right atrial volumes were markedly enlarged. Detailed baseline characteristics and results of univariable Cox regression analyses for mortality are summarized in Table 1, and comprehensive echocardiographic measurements are presented in Table 2. Among RV-centered echocardiographic parameters, greater TVAPD (HR, 1.14; 95% CI, 1.01–1.28; *p* = 0.030) and increased RVLD (HR, 1.11; 95% CI, 1.01–1.21; *p* = 0.019) were significantly associated with all-cause mortality; NYHA class IV also conferred higher clinical risk (HR, 6.50; 95% CI, 1.40–38.90; *p* = 0.015).

### 3.2. Day Major Adverse Events and Exploratory Imaging Models

Within 30 days after TTVR, 14 patients (22.6%), including three deaths, experienced MAEs. Significant intraoperative or postoperative hemorrhage occurred in eight patients, seven of whom required surgical re-exploration via sternotomy. In addition, one patient underwent repeat open-heart tricuspid valve replacement, and six patients required permanent pacemaker implantation for newly developed atrioventricular conduction block. Because this composite endpoint was dominated by perioperative or device-related complications, RVFWS, RVLD, RVESVI, and TVAPD were retained only as exploratory imaging variables in subsequent models (Supplemental Table S1).

Firth logistic regression analyses are summarized in Table 3. Although larger RVESVI, RVLD, and TVAPD showed associations in RV-focused exploratory models, the 30-day MAE endpoint was dominated by perioperative or device-related complications and estimates were imprecise because of the small number of events. Accordingly, these models should be interpreted as hypothesis-generating rather than as evidence that baseline RV dysfunction or remodeling directly caused early MAEs.

Exploratory sPAP threshold analyses using >35, >45, and >50 mmHg cutoffs were incorporated into the revised analysis plan because pulmonary pressure and RV-pulmonary arterial coupling are directly relevant to RV reserve in severe TR. These thresholds should be interpreted with caution and validated with patient-level data in larger cohorts.

Reviewer-requested exploratory threshold analyses for 30-day MAEs are presented in Table 3. TAPSE < 15 mm showed a signal in this exploratory analysis (OR, 3.53; 95% CI, 1.02–12.24; *p* = 0.047), whereas TAPSE < 17 mm showed a nonsignificant trend (OR, 2.36; 95% CI, 0.63–8.89; *p* = 0.203). The corresponding exploratory estimates were as follows: RVFWS < 19% (OR, 0.99; 95% CI, 0.28–3.55; *p* = 0.992), RVFWS < 16% (OR, 1.92; 95% CI, 0.51–7.24; *p* = 0.336), 3D RVEF < 45% (OR, 2.60; 95% CI, 0.46–14.81; *p* = 0.282), 3D RVEF < 35% (OR, 1.09; 95% CI, 0.04–28.28; *p* = 0.958), RV-FAC < 35% (OR, 3.96; 95% CI, 0.79–19.93; *p* = 0.095), RV-FAC < 25% (OR, 3.34; 95% CI, 0.06–176.10; *p* = 0.550), RV basal diameter > 50 mm (OR, 0.48; 95% CI, 0.11–2.11; *p* = 0.329), and RV basal diameter > 55 mm (OR, 0.61; 95% CI, 0.10–3.94; *p* = 0.608). These estimates should be interpreted cautiously because several thresholds produced sparse cells and the early endpoint was dominated by procedural complications.

**Figure 4:**
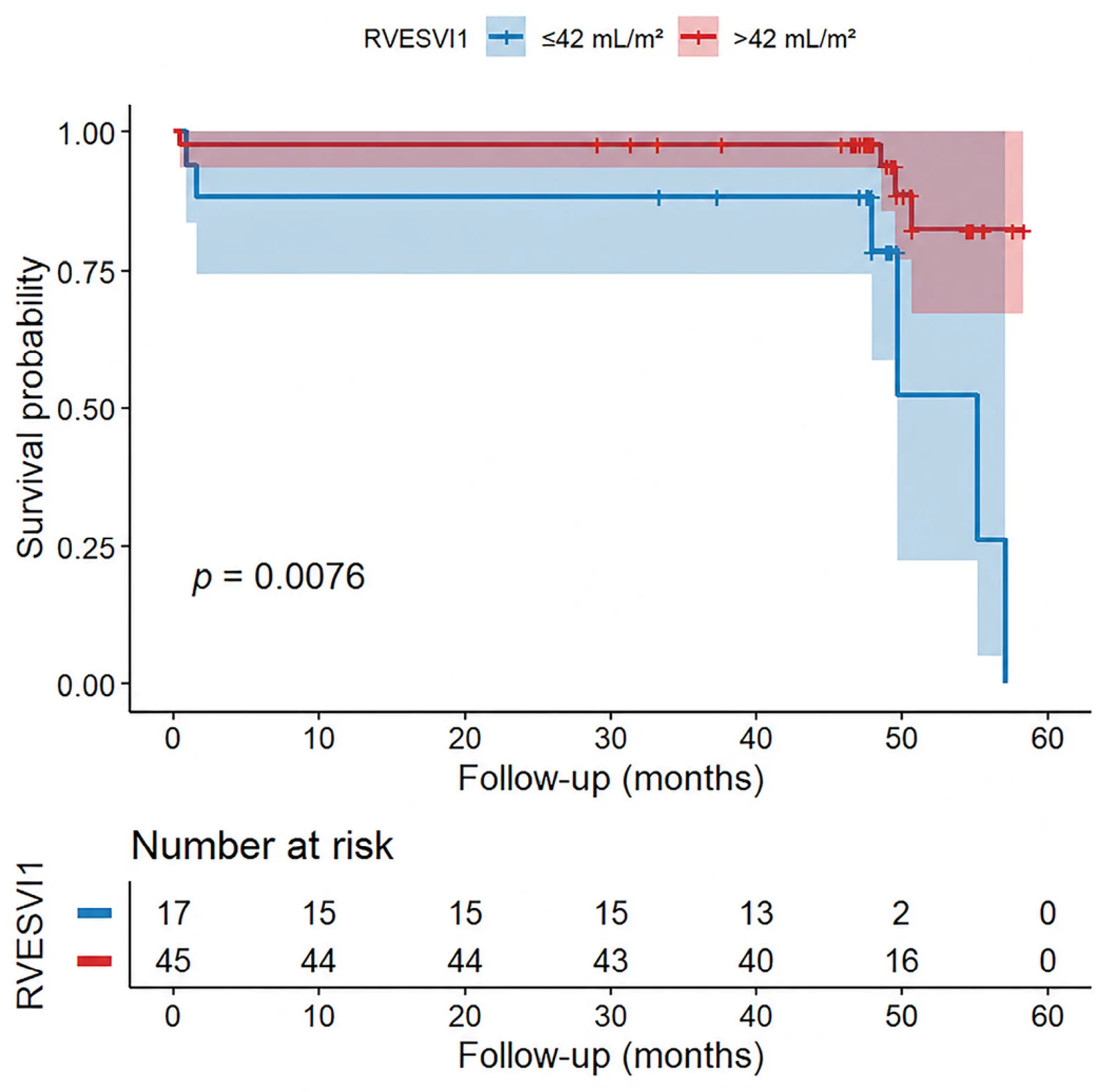
Kaplan–Meier curves stratified by RVESVI > 42 mL/m^2^.

**Figure 5:**
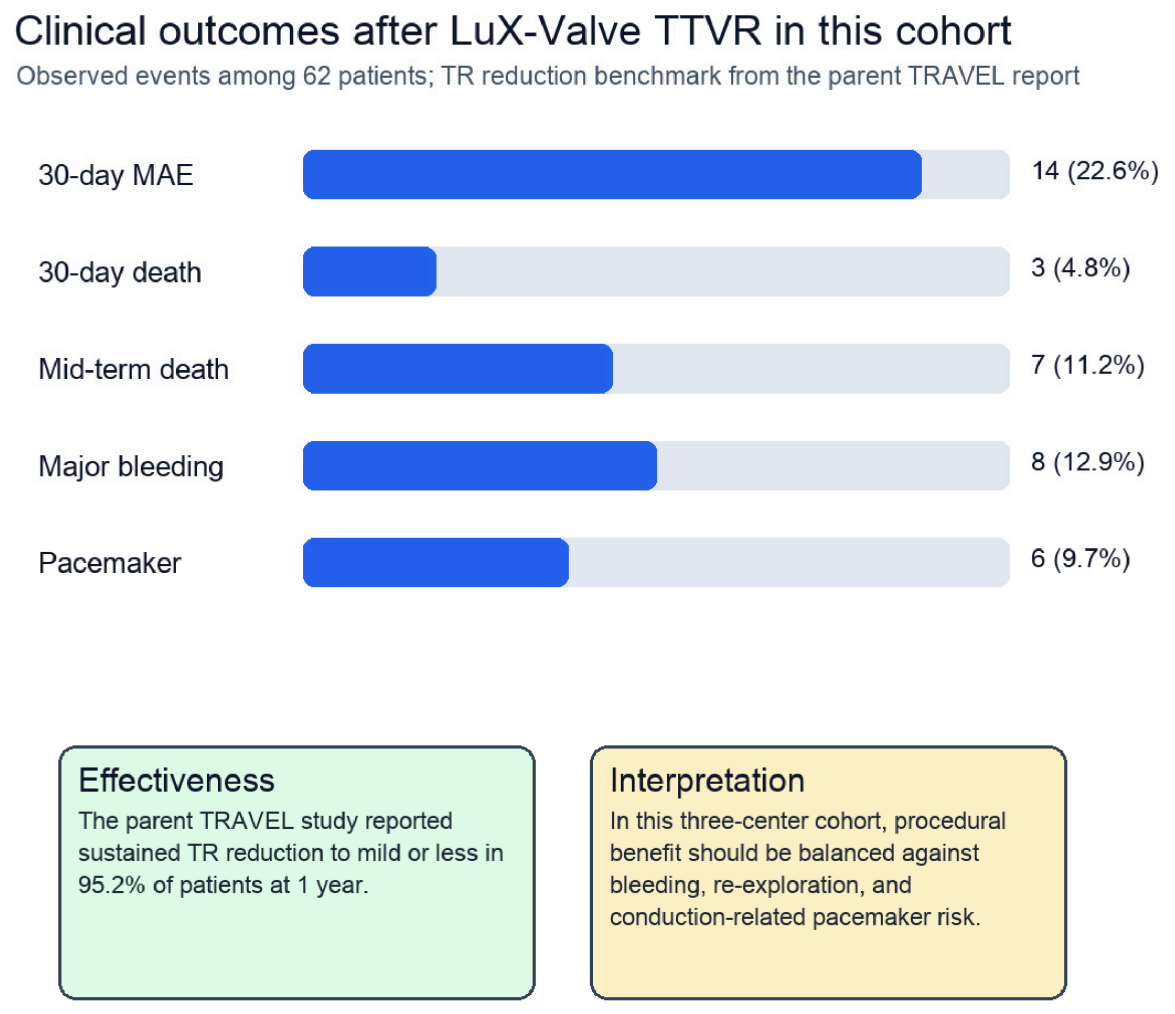
Outcomes and early safety profile after LuX-Valve TTVR in the present cohort, including 30-day MAEs, mortality, bleeding, and pacemaker implantation, with contextual TR reduction from the parent TRAVEL study.

The RVESVI-based risk stratification is presented as a hypothesis-generating analysis and should be considered alongside the procedural composition of 30-day MAEs. Kaplan–Meier analysis stratified by RVESVI > 42.0 mL/m^2^ showed significantly higher event rates (log-rank *p* = 0.007) (Figure 4). Procedural effectiveness and complications of LuX-Valve TTVR are summarized in Table 4 and Figure 5.

### 3.3. LuX-Valve Procedural Outcomes and Valve-Specific Effectiveness (Table 5)

All patients underwent orthotopic TTVR with the LuX-Valve system. The device is designed as a radial force-independent tricuspid prosthesis, using an atrial disc, leaflet graspers, and an interventricular septal anchor for fixation. In this cohort, the mean follow-up was 46.2 months, with seven all-cause deaths (11.2%) and three deaths within 30 days (4.8%). The principal early complications were bleeding requiring intervention, surgical re-exploration, repeat tricuspid valve replacement, and new permanent pacemaker implantation. These outcomes highlight the need to report procedural benefit together with safety events when applying LuX-Valve TTVR in high-risk severe TR.

The effectiveness endpoint was clinically centered on elimination or durable reduction of severe TR after valve replacement. In the parent TRAVEL report, LuX-Valve implantation achieved 100% device success, 98.4% procedural success, and sustained reduction of TR to mild or less in 95.2% of patients at 1 year [7]. The present three-center analysis should therefore be interpreted as a mid-term prognostic extension of a procedure with established short-term TR reduction, while acknowledging that center-specific follow-up TR grades should be reported when available.

### 3.4. Reproducibility

The interobserver and intraobserver ICC were 0.88 and 0.91, respectively, for RVESVI. Reproducibility for RVFWS and other RV functional indices should be reported in future analyses to support broader clinical implementation.

## 4. Discussion

In this multicenter study of 62 patients undergoing TTVR with the LuX-Valve system, we summarized mid-term outcomes, perioperative safety, procedural effectiveness, and exploratory RV-centered imaging signals. The principal findings were as follows: (1) LuX-Valve TTVR achieved favorable mid-term survival and established procedural effectiveness in the TRAVEL context; (2) 30-day MAEs occurred in 14 patients (22.6%) and were mainly composed of perioperative bleeding, surgical re-exploration, repeat intervention, and conduction-related pacemaker implantation; and (3) RV remodeling and functional markers showed exploratory associations but should not be interpreted as demonstrating that baseline RV dysfunction or remodeling directly drove 30-day MAEs. These results support reporting early safety and procedural effectiveness separately from hypothesis-generating imaging risk stratification.

### 4.1. Mid-Term Outcome of TTVR

For patients with severe TR, TTVR has been shown to be superior to optimal medical therapy alone in reducing cardiac mortality and heart failure hospitalization at 1 year, as demonstrated in recent trials such as TRAVEL and TRISCEND [4,5]. In pairwise comparisons, patients undergoing valve replacement consistently achieved more favorable outcomes than controls, including a lower incidence of all-cause mortality [4,5]. Compared with optimal medical therapy alone, treatment with TTVR also resulted in substantial improvements in symptoms, functional capacity, and quality of life [6]. With the rapid evolution of the TTVR field and an expanding evidence base, the first device has already received CE Mark and U.S. Food and Drug Administration approval, with commercial implantation now underway [17].

At this stage, longer-term follow-up is particularly important to determine the durability and prognostic impact of TTVR in patients with severe TR. In the present study, we analyzed the cohort from three high-volume centers that participated in the TRAVEL study initiated in 2020 [5], with a mean follow-up of 3.8 years. The overall mortality rate was 11.2%, consistent with previously reported 1-year mortality rates, thereby providing the first evidence that mid-term outcomes after TTVR are favorable. Notably, the 1-year mortality in our cohort was only 4.8%, substantially lower than the 10.3% previously reported in the overall TRAVEL cohort of 126 patients across eight centers. This difference likely reflects the inclusion of more experienced, higher-volume centers in the current analysis, underscoring that outcomes after TTVR are not uniform and may be strongly influenced by institutional expertise and operator experience. This selection bias limits the generalizability of our findings to lower-volume centers or non-trial populations.

### 4.2. Prognostic Stratification

Although the feasibility, safety, and symptomatic benefits of TTVR in patients with severe TR have been demonstrated in multicenter studies, prognostic stratification beyond procedural success has remained limited. Imaging-based markers to guide patient selection and postoperative monitoring have not been well established. Our findings extend this evidence by emphasizing RV-centered markers, especially RVESVI, RVLD, TVAPD, RVFWS, TAPSE, RV-FAC, S-TDI, sPAP, and TAPSE/sPAP, rather than left-sided parameters. For 30-day MAEs, however, these imaging models should be interpreted cautiously because the endpoint was largely procedural or device-related. RVESVI > 42.0 mL/m^2^ emerged as a hypothesis-generating cutoff that may be incorporated into routine preoperative echocardiographic assessment for further investigation in larger cohorts. The additional sPAP thresholds of >35, >45, and >50 mmHg were added because pulmonary vascular load is central to RV reserve and post-procedural adaptation. Additional reviewer-requested RV functional thresholds were incorporated to improve clinical interpretability, but only TAPSE < 15 mm reached statistical significance in this exploratory threshold analysis.

### 4.3. Pathophysiological Insights

The prognostic value of RV remodeling in this study is biologically plausible. In severe TR, chronic volume overload promotes progressive RV dilation, annular enlargement, leaflet malcoaptation, and impaired contractile reserve. RVESVI reflects the burden of maladaptive remodeling at end systole, whereas RVLD and TVAPD capture chamber and annular geometry that may influence both procedural complexity and post-procedural adaptation. RVFWS, TAPSE, RV-FAC, S-TDI, and TAPSE/sPAP provide complementary information about RV systolic function and RV-pulmonary arterial coupling.

Successful TR reduction abruptly changes RV loading conditions. Patients with advanced RV dilation, impaired contractile reserve, or elevated sPAP may be vulnerable to afterload mismatch, low cardiac output, and bleeding or hemodynamic instability after valve implantation. For this reason, RV-pulmonary interaction is more clinically actionable than left-sided strain alone in patients selected for TTVR. The revised analysis therefore prioritizes RV parameters and pulmonary pressure thresholds when assessing worse outcomes. The LuX-Valve system may be particularly relevant in this anatomy because its fixation strategy is radial force-independent. The combination of an atrial disc, leaflet graspers, and an interventricular septal anchor is intended to stabilize the prosthesis in a dilated tricuspid annulus without excessive radial force. This design may broaden treatment options for high-risk patients with severe TR, but it also requires careful imaging guidance, septal assessment, and post-release evaluation for residual TR, valve gradient, bleeding, and conduction disturbance.

In contrast to analyses centered on LV-GLS, RVESVI directly reflects chronic volume overload and maladaptive RV remodeling resulting from longstanding severe TR. Persistent regurgitant flow leads to progressive dilation, increased wall stress, and impaired contractile efficiency. An enlarged RVESVI indicates reduced ventricular reserve and heightened vulnerability to abrupt hemodynamic shifts once regurgitation is eliminated by TTVR [18,19]. The sudden reduction in RV preload, coupled with increased effective forward afterload, may precipitate acute afterload mismatch, presenting as low cardiac output and RV failure. Marked RV enlargement may also increase the technical difficulty of anchoring the LuX-Valve device and heighten the risk of bleeding.

## 5. Limitations

This study has several limitations. First, the sample size was relatively small, with only 62 patients across three centers, and the number of clinical events was limited (seven deaths and 14 30-day MAEs). This significantly limits statistical power and introduces a high risk of model overfitting, even with the use of Firth’s penalized regression; the wide confidence intervals for certain predictors reflect this inherent statistical uncertainty. Second, this is a post hoc analysis of a single-arm clinical trial enrolling a highly selected patient group from three high-volume centers, which introduces selection bias and limits generalizability to broader clinical practice. Third, echocardiographic examinations were performed at each participating center, and although analyses were conducted by an experienced observer, inter-institutional variability cannot be completely excluded. Fourth, RV remodeling, RV functional indices, sPAP, and TR reduction were assessed using available baseline and follow-up data; longitudinal imaging follow-up with standardized core-laboratory adjudication would be necessary to more accurately capture reverse remodeling after TTVR. Fifth, the small number of events precluded internal validation techniques and robust sensitivity analyses of individual components of the composite MAE endpoint, including sPAP threshold analyses. Future prospective, large-scale multicenter studies with standardized imaging protocols, longer follow-up, and external validation are warranted to confirm RV-focused predictors and to establish validated risk scores for clinical practice. The newly added RV functional threshold estimates were exploratory and were limited by sparse event counts for several cutoffs.

## 6. Conclusions

LuX-Valve TTVR showed favorable mid-term survival and clinically meaningful procedural effectiveness, with 30-day MAEs mainly reflecting perioperative or device-related complications. RV-centered echocardiographic parameters, including RVESVI, RVLD, TVAPD, RV functional indices, sPAP thresholds, and RV-pulmonary arterial coupling, remain hypothesis-generating markers for risk stratification and require validation in larger cohorts. TAPSE, RVFWS, 3D RVEF, RV-FAC, and RV basal diameter threshold findings also require validation in larger cohorts.

## Figures and Tables

**Table 1:** Baseline characteristics and univariate COX analysis.

Variable	Value	HR	Lower 95%	Upper 95%	*p*
Clinical					
age (y)	66.5 (61.0–72.8)	1.11	0.99	1.23	0.054
female n (%)	52 (83.8)	0.46	0.09	2.34	0.355
BMI (kg/m^2^)	23.6 (21.3–25.5)	1.00	0.79	1.26	0.983
HP n (%)	16 (25.8%)	0.63	0.09	4.21	0.590
DM n (%)	11 (17.7%)	0.29	0.01	6.16	0.310
Hyperlipidemia n (%)	4 (6.5%)	0.91	0.04	19.42	0.950
stroke n (%)	2 (3.2%)	7.77	1.15	52.52	0.070
Ascites n (%)	5 (8.1%)	3.84	0.18	81.96	0.440
NYHA					
class III n (%)	50(80.6)				
class IV n (%)	12 (19.4)	6.50	1.40	28.90	0.015
AF n (%)	58 (93.6%)				
History of OR type		0.53	0.10	2.88	0.390
MVR n (%)	26 (41.9)				
BVR n (%)	13 (21.0)				
CABG n (%)	2 (3.2)				
Pacemaker	6 (9.6)	0.56	0.00	1923.28	0.563
Albumin (g/L)	42.85 (39.7–44.6)	0.85	0.74	0.98	0.057
Creatinine (μmol/L)	79.28 (64.1–85.8)	1.09	0.97	1.24	0.140
NT-proBNP (pg/mL)	528.0 (338.0–1026.0)	1.00	1.00	1.00	0.923
TTVR duration (min)	150.5 (115.3–203.0)	1.00	1.00	1.01	0.150
VT (hour)	24.0 (18.0–40.0)	1.00	0.99	1.01	0.928

BMI, Body Mass Index; HP, Hypertension; DM, Diabetes Mellitus; NYHA, New York Heart Association functional class; AF, Atrial Fibrillation; MVR, Mitral Valve Replacement; BVR, Bicuspid/Double Valve Replacement; CABG, Coronary Artery Bypass Grafting; Pacemaker, NT-proBNP, N-terminal pro-B-type Natriuretic Peptide; TTVR, Transcatheter Tricuspid Valve Replacement; VT, Ventilation time.

**Table 2:** Baseline echocardiographic measurements and univariate COX analysis.

Variable	Value	HR	Lower 95%	Upper 95%	*p*
TR grade		0.62	0.14	2.78	0.505
4 n (%)	28 (45.2%)				
≥5 n (%)	34 (54.8%)				
LAEDD (mm)	53.0 (45.3–58.8)	1.05	0.96	1.16	0.250
LVEF (%)	63.5 (58.0–70.8)	1.06	0.98	1.15	0.152
LV-GLS (%)	18.0 (21.8–15.3)	1.49	1.15	1.93	0.000
RVOTEDD	35.0 (30.0–39.3)	1.05	0.93	1.20	0.421
TAPSE (mm)	15.9 (12.5–18.3)	0.99	0.86	1.15	0.908
RV-FAC (%)	47.8 (41.9–52.3)	0.97	0.89	1.06	0.500
TVAPD (mm)	44.7 (41.7–48.0)	1.14	1.01	1.28	0.030
TVA (mm^2^)	1506.0 (1346.8–1757.3)	1.03	0.99	1.07	0.189
RVLD (mm)	65.0 (56.9–71.6)	1.11	1.01	1.21	0.019
RVEDVI (mL/m^2^)	71.6 (56.9–98.4)	1.01	0.99	1.02	0.602
RVESVI (mL/m^2^)	31.4 (24.9–43.4)	1.01	0.97	1.05	0.638
LVEF-3D (%)	53.7 (51.3–57.8)	1.01	0.92	1.12	0.810
RVFWS (%)	25.2 (22.0–32.8)	1.01	0.97	1.07	0.610
RAVI (mL/m^2^)	87.3 (57.0–137.7)	1.01	1.00	1.01	0.166
LAVI (mL/m^2^)	85.9 (52.3–121.3)	1.00	1.00	1.01	0.523
IVC (mm)	25.0 (20.0–28.8)	1.06	0.94	1.20	0.316
TR velocity (m/s)	2.6 (2.30–2.90)	1.19	0.23	6.27	0.836
LASr_ED (%)	5.5 (3.80–9.00)	0.99	0.86	1.13	0.837
RASr_ED (%)	9.10 (4.20–12.30)	1.06	0.99	1.14	0.149
RV-PA coupling (cm/mmHg)	0.5 (0.4, 0.8)	0.47	0.03	7.42	0.588

TTVR, Transcatheter Tricuspid Valve Replacement; TR, Tricuspid Regurgitation; LAEDD, Left Atrial End-Diastolic Diameter; LVEF, Left Ventricular Ejection Fraction; LV-GLS, Left Ventricular Global Longitudinal Strain; RVOTEDD, Right Ventricular Outflow Tract End-Diastolic Diameter; TAPSE, Tricuspid Annular Plane Systolic Excursion; RV-FAC, Right Ventricular Fractional Area Change; TVAPD, Tricuspid Valve Annular Plane Displacement; TVA, Tricuspid Valve Annulus area; RVLD, Right Ventricular Long-axis Dimension; RVEDVI, Right Ventricular End-Diastolic Volume Index; RVESVI, Right Ventricular End-Systolic Volume Index; LVEF-3D, Left Ventricular Ejection Fraction by 3D echo; RVFWS, Right Ventricular Free Wall Strain; RAVI, Right Atrial Volume Index; LAVI, Left Atrial Volume Index; IVC, Inferior Vena Cava diameter; LASr_ED, Left Atrial Strain during reservoir phase at End-Diastole; RASr_ED, Right Atrial Strain during reservoir phase at End-Diastole; RV-PA coupling, Right Ventricular–Pulmonary Artery coupling (TAPSE/PASP).

**Table 3:** Exploratory Firth logistic regression models for RV-focused variables and 30-day complications after TTVR.

Model	RV-Focused Variable	OR (95% CI)	*p* Value
Model 1	RVESVI	1.38 (1.08–1.85)	0.021
Model 2	RVLD	1.25 (1.09–2.28)	0.005
Model 3	TVAPD	1.18 (1.03–2.11)	0.003
Model 4	RVFWS	1.15 (1.02–1.98)	0.011
Model 5	sPAP > 35 mmHg	1.08 (0.78–1.23)	0.067
Model 6	sPAP > 45 mmHg	1.17 (1.08–2.23)	0.005
Model 7	sPAP > 50 mmHg	1.47 (1.09–2.67)	0.031
Model 8	TAPSE < 17 mm	2.36 (0.63–8.89)	0.203
Model 9	TAPSE < 15 mm	3.53 (1.02–12.24)	0.047
Model 10	RVFWS < 19%	0.99 (0.28–3.55)	0.992
Model 11	RVFWS < 16%	1.92 (0.51–7.24)	0.336
Model 12	3D RVEF < 45%	2.60 (0.46–14.81)	0.282
Model 13	3D RVEF < 35%	1.09 (0.04–28.28)	0.958
Model 14	RV-FAC < 35%	3.96 (0.79–19.93)	0.095
Model 15	RV-FAC < 25%	3.34 (0.06–176.10)	0.550
Model 16	RV basal diameter > 50 mm	0.48 (0.11–2.11)	0.329
Model 17	RV basal diameter > 55 mm	0.61 (0.10–3.94)	0.608

**Table 4:** LuX-Valve procedural outcomes, effectiveness, and early safety profile.

Endpoint	Definition/Time Point	No./Value	Rate	Clinical Relevance
Completed LuX-Valve TTVR	Index procedure in this cohort	62	100%	Base population for outcome analysis
Mean follow-up	Mid-term observation	46.2 months	100%	Durability window for survival assessment
30-day MAE	Death or device-related complication	14	22.6%	Primary early safety endpoint
30-day death	All-cause death within 30 days	3	4.8%	Early mortality signal
Mid-term death	All-cause death during follow-up	7	11.2%	Primary mid-term outcome
Major bleeding	Intra-/post-operative hemorrhage	8	12.9%	Key LuX-Valve safety issue
Surgical re-exploration	Sternotomy after bleeding	7	11.3%	Procedure-related morbidity
Repeat TV replacement	Open-heart tricuspid valve replacement	1	1.6%	Unplanned reintervention
Permanent pacemaker	New AV block after TTVR	6	9.7%	Conduction complication
TR reduction	Parent TRAVEL 1-year benchmark	TR mild or less	95.2%	Effectiveness context for LuX-Valve

**Table 5:** LuX-Valve device characteristics, implantation technique, and potential benefits compared with other transcatheter tricuspid approaches.

Feature	LuX-Valve	Potential Benefit	Procedural Consideration	Comparison Point
Access/position	Orthotopic tricuspid replacement	Eliminates severe TR at valve level	Requires coaxial alignment and imaging guidance	Replacement rather than leaflet repair
Fixation	Atrial disc, leaflet graspers, septal anchor	Radial force-independent stability	Septal thickness and anchor trajectory must be assessed	Differs from devices relying mainly on annular radial force
Valve tissue	Tri-leaflet bovine pericardial valve	Restores forward flow and reduces regurgitant volume	Post-release gradient and residual TR must be checked	Bioprosthetic valve function
Imaging guidance	TTE/TEE/CT planning with intraprocedural guidance	Improves sizing, depth, leaflet capture and release	Operator and center experience are important	Multimodality workflow
Safety profile	Bleeding, re-exploration, pacemaker risk	Clarifies patient counseling and monitoring	Balance effectiveness with procedural risk	Report outcomes with complications

RV-focused exploratory models were evaluated with Firth logistic regression because of the limited number of 30-day MAEs. OR, odds ratio; CI, confidence interval; other abbreviations are as Table 2. Reviewer-requested threshold rows were calculated as exploratory binary threshold models; RVFWS threshold models included 60 patients with available strain data, and S’ threshold models were not estimated because tissue Doppler S’ was unavailable.

## Data Availability

The data presented in this study are available on request from the corresponding authors. The data are not publicly available due to restrictions concerning patient privacy and confidentiality.

## Supplementary Materials

The following supporting information can be downloaded at: https://www.mdpi.com/article/10.3390/jcdd13080381/s1, Table S1: Univariable analyses for predictors of 30-day complications after TTVR.

## Abbreviations

The following abbreviations are used in this manuscript:

CI	confidence interval
OR	odds ratio
HR	hazard ratio
MAE	major adverse event
NYHA	New York Heart Association
HFpEF	heart failure with preserved ejection fraction
RV	right ventricle/ventricular
LV-GLS	left ventricular global longitudinal strain
RV-GLS	right ventricular global longitudinal strain
RVESVI	right ventricular end-systolic volume index
RVLD	right ventricular longitudinal dimension
TAPSE	tricuspid annular plane systolic excursion
TTE	transthoracic echocardiography
TVAPD	tricuspid annular anterior–posterior diameter
TTVR	transcatheter tricuspid valve replacement
TR	tricuspid regurgitation
TRAVEL	Transcatheter Right Atrial-Ventricular Valve Replacement With LuX-Valve
ICC	intraclass correlation coefficients
