# Supplementary material for: Mid-Term Outcomes and Echocardiographic Predictors of TTVR: Insights from the TRAVEL Study
Source: J Cardiovasc Dev Dis. 2026 Aug 11;13(8):381. doi: 10.3390/jcdd13080381 (PMC13513638; doi:10.3390/jcdd13080381)
Supplement: Supplementary file 1 [file jcdd-13-00381-s001.zip › jcdd-4265440-supplementary.pdf]

**Supplemental table S1 Univariable analyses for predictors of 30-day complications after TTVR.**

| variate | OR (95% CI) | P |
|---|---|---|
| age | 1.050 (0.962–1.150) | 0.275 |
| BMI | 0.791 (0.593–1.008) | 0.059 |
| HP | 0.382 (0.039–1.889) | 0.259 |
| DM | 1.347 (0.227–5.970) | 0.716 |
| Hyperlipidemia | 2.233 (0.200–15.554) | 0.461 |
| stroke | 0.962 (0.007–13.015) | 0.980 |
| Ascites | 16.579 (0.822–2495.974) | 0.066 |
| NYHA IV | 3.624 (0.847–14.959) | 0.081 |
| no surgery | 0.404 (0.068–1.815) | 0.244 |
| BVR | 0.686 (0.111–3.243) | 0.644 |
| CABG | 0.631 (0.004–9.189) | 0.766 |
| Albumin | 1.016 (0.877–1.212) | 0.838 |
| Creatinine | 0.965 (0.919–1.004) | 0.084 |
| NT.proBNP | 0.999 (0.997–1.000) | 0.227 |
| LAEDD | 0.983 (0.918–1.049) | 0.601 |
| LVEF | 0.996 (0.926–1.071) | 0.909 |
| LVGLS | 1.300 (1.055–1.601) | 0.016 |
| TAPSE | 0.942 (0.809–1.075) | 0.394 |
| RV-FAC | 0.957 (0.877–1.039) | 0.301 |
| RVLD | 1.126 (1.013–1.251) | 0.028 |
| TVAPD | 1.144(1.00,1.31) | 0.048 |
| RVEDV | 1.010 (0.999–1.023) | 0.078 |
| RVEDVI | 1.015 (0.999–1.034) | 0.064 |
| RVESV | 1.026 (1.002–1.053) | 0.031 |
| RVESVI | 1.038 (1.005–1.078) | 0.026 |
| EF-3D | 0.981 (0.902–1.073) | 0.667 |
| RVFWS | 1.082 (1.027–1.140) | 0.003 |
| RAVI | 1.005 (0.996–1.013) | 0.281 |
| LAVI | 1.002 (0.994–1.009) | 0.514 |
| RASr_ED | 0.988 (0.873–1.078) | 0.805 |
| TRgrade=4 | 0.503 (0.112–1.901) | 0.317 |
| RV-PA Coapling | 0.103(0.005,2.371) | 0.156 |

Abbreviations are as table1 and 2.
